# Correction: *Congenital sucrase-isomaltase mutations worsen IBS-linked V15F dysfunction and trafficking*

**DOI:** 10.1136/gutjnl-2025-336813corr1

**Published:** 2026-02-19

Tannous S, Husein DM, Naim HY. Congenital sucrase-isomaltase mutations worsen IBS-linked V15F dysfunction and trafficking. *Gut* Published Online First: 21 October 2025.

Figure 1A was incorrectly labelled in the right panel, where the band marked 250 kDa represents 150 kDa. The labelling has been corrected in the published version doi:10.1136/gutjnl-2025-336813corr1

